# Transaminitis among Patients with Dengue Fever Visiting a Tertiary Care Centre

**DOI:** 10.31729/jnma.8258

**Published:** 2023-09-30

**Authors:** Rabindra Jang Rayamajhi, Sangita Thapa, Prachi Rayamajhi, Suman Maharjan, Roshan Kumar Ray Yadav, Kumar Roka

**Affiliations:** 1 Department of Internal Medicine, Shree Birendra Hospital, Chhauni, Kathmandu, Nepal; 2 Department of Biochemistry, Richmond Gabriel University, Arnos Vale, Saint Vincent and the Grenadines; 3 Department of Biochemistry, Shree Birendra Hospital, Chhauni, Kathmandu, Nepal; 4 Shree Birendra Hospital, Chhauni, Kathmandu, Nepal

**Keywords:** *alanine transaminase*, *aspartate transaminase*, *dengue*

## Abstract

**Introduction:**

Transaminitis is a condition where serum aspartate transaminase and alanine transaminase increase indicating liver dysfunction. One such disease where liver involvement might be observed is dengue, which is a mosquito-borne viral infection. The aim of the study was to find out the prevalence of transaminitis among patients with dengue fever in a tertiary care centre.

**Methods:**

A descriptive cross-sectional study was conducted in a tertiary care centre from 1 November 2022 to 31 March 2023 after obtaining ethical approval from the Institutional Review Committee. Informed written consent was taken before collecting the data. A rapid immunochromatography test was used to confirm dengue infection. Serum aspartate transaminase and alanine transaminase were measured through routine Reitman and Frankel's enzymatic method. Dengue-confirmed patients from the medical outpatient department, fever clinic, and medical ward of the centre were included in the study. Patients with known prior liver diseases or any other chronic diseases, pregnancy, and patients in the hospice unit were excluded. Convenience sampling method was used. The point estimate was calculated at a 95% Confidence Interval.

**Results:**

Among 442 dengue infected patients, the prevalence of transaminitis was 188 (42.53%) (37.92-47.13, 95% Confidence Interval). The highest frequency of dengue positive was observed among the 18-35 years age group, which was 97 (51.59%) with male predominance 134 (71.27%).

**Conclusions:**

The prevalence of transaminitis among patients with dengue fever in a tertiary care centre was found to be lower than other studies done in similar settings.

## INTRODUCTION

Transaminitis is a condition where serum aspartate transaminase (AST) and alanine transaminase (ALT) increase. The serum transaminases increase when the hepatocytes are damaged during different diseases, and thus transaminases may act as a surrogate marker for assessing the hepatic involvement with the severity of different diseases.^1^

Dengue fever is more prevalent in Southeast Asia, which is transmitted by *Aedes aegypti.* It is usually self-limiting with flu-like symptoms but severe dengue might occur when the previously infected person with one dengue virus serotype gets reinfected with another dengue serotype, and patients may develop complications like bleeding, endothelial leakage, and organ involvement like cardiomyopathy, encephalopathy, and hepatic injury.^2,3^ In dengue, along with other clinical features, transaminitis suggests hepatic involvement.^4^ Serum AST is usually elevated more than ALT in dengue during the early days of infection.^5^

The aim of the study was to find out the prevalence of transaminitis among patients with dengue fever in a tertiary care centre.

## METHODS

This descriptive cross-sectional study was conducted on the patients presented to Shree Birendra Hospital, Chhauni, Kathmandu, Nepal from 1 November 2022 to 31 March 2023, and the ethical approval was obtained from the Institutional Review Committee (Reference number: 245). Patients diagnosed with dengue were enrolled in the study after their informed written consent. Dengue-confirmed patients from the medical outpatient department (OPD), fever clinic, and admitted patients in the medical ward of the centre with complete data were included in the study. Patients with known prior liver diseases or any other chronic diseases, pregnancy, and patients in the hospice unit were excluded. Convenience sampling method was used. The sample size was calculated using the following formula:


$$\begin{array}{ll} \text{n} & ={\text{Z}}^{2}\times \frac{\text{p}\times \text{q}}{{\text{e}}^{\text{2}}}\\ & =1.96^{2}\times \frac{0.50\times 0.50}{0.05^{2}}\\ & =385 \end{array}$$

Where,

n = minimum required sample size
Z = 1.96 at 95% Confidence Interval (CI)
p = prevalence taken as 50% for maximum sample size calculation
q = 1-p
e = margin of error, 5%

The minimum required sample size was 385. Adding 15% non-response rate, the final sample size taken was 442.

Blood samples of dengue-confirmed patients were collected, and serum was immediately separated. This study used a rapid test based on the immunochromatography principle to detect NS1 Ag, IgM, and IgG. NS 1 Ag and IgM positive (either one or both) confirms dengue infection and is consistent with acute phase infection. NS 1 was detected on day 1 of exposure, and IgM on 3 to 7 days following dengue infection. After 14 days following exposure, all patients would have developed IgG antibodies to the dengue virus, which persists throughout their life, and thus, detection of only IgG signifies the patient had dengue infection in the past.^6,7^ Serum AST and ALT were measured in Cobas fully automated analyzer through routine Reitman and Frankel's enzymatic method.^8^ For diagnosis of transaminitis, serum AST and ALT levels should be greater than 40 U/L based on the hospital laboratory reference ranges.

Data were entered in Microsoft Excel 2016 and analysed using IBM SPSS statistics version 23.0. The point estimate was calculated at a 95% CI.

## RESULTS

Out of 442 dengue patients, transaminitis was found in 188 (42.53%) (37.92-47.13, 95% CI). Increased serum AST was observed in 233 (52.71%) and ALT was seen in 204 (46.15%). The highest frequency of transaminitis among dengue positive was observed among 18-35 years of age group, which was 97 (51.59%) with male predominance 134 (71.27%) (Table 1).

**Table 1:** Distribution of transaminitis among dengue patients based on demographic factors (n= 188).

Age group (years)	n (%)
<18	6 (3.19)
18-35	97 (51.59)
36-55	41 (21.80)
56-75	22 (11.70)
>75	1 (0.53)
**Gender**	
Male	134 (71.27)
Female	54 (28.72)

Among transaminitis patients, 71(37.76%) and 54(28.72%) complained symptomsof nausea andvomiting, respectively. Decreased platelet count wasseen among 151 (80.31%) of the patients. Increaseddirect bilirubin level was seen in 109 (57.97%) (Table 2).

**Table 2:** Clinical symptoms and laboratory parameters in transaminitis among dengue patients (n= 188).

Clinical symptoms	n (%)
Nausea	71 (37.76)
Vomiting	54 (28.72)
Hepatomegaly	2 (1.06)
Petechia/purpura	11 (5.85)
**Laboratory findings**	
Low platelet count	151 (80.31)
Increased creatinine	27 (14.36)
Increased total serum bilirubin	29 (15.42)
Increased direct serum bilirubin	109 (57.97)

## DISCUSSION

The prevalence of transaminitis was 188 (42.53%) among the total participants, and the elevation of AST was more than ALT. This finding, 46.30% is lower than in other study.^3^ In other similar studies, it was found that, the abnormal serum aminotransferases had a similar trend of increase in AST/ALT in dengue fever but with higher frequency than our population.^4,7^

Transaminitis in dengue infection could be due to the direct dengue virus effect on the hepatocytes or the host's immune response to the viral infection. The receptors present on hepatocytes and Kupffer cells are the primary targets of the dengue virus to which they attach and infect the cells. This causes cellular apoptosis due to viral cytopathy, mitochondrial dysfunction, immune response and accelerated endoplasmic reticular stress.^9-11^

Severe dengue may be due to exaggerated immune reactions to recurrent infections. Infection with dengue virus stimulates a cytokine storm often called a cytokine Tsunami causing the concentrations of different cytokines like interleukins, tumor necrosis factor-α, and interferon-γ to reach peaks.^12^ The higher level of AST compared to ALT could be due to the release of the enzymes from the damaged myocytes, which helps to differentiate dengue infection from other acute hepatitis caused by Hepatitis A, B, and C because of their reverse AST/ALT pattern.^1,5^

Jaundice is linked with severe hepatic disease and is a poor prognostic factor in itself. In our study, hyperbilirubinemia was seen in 15.42% with increased direct bilirubin in 57.97% of the cases. Some studies noted a higher frequency of hyperbilirubinemia in dengue infection while others had findings similar to the present study.^12,13^ High bilirubin may act as a poor prognostic marker in dengue infection. Furthermore, hepatomegaly was seen only in 1.06% of cases. This finding is low as compared to other studies where the frequency of hepatomegaly in adult dengue patients was much higher.^14^

This study has certain limitations. Due to cost factors and unavailability in our setup, we could not measure the types of dengue virus. Since this is a cross-sectional study, we could not follow up on whether the serum transaminases declined with the improvement of acute dengue infection. The study recruited patients from a single tertiary centre, so the results may not be implied to the whole population. Further, an analytical investigation needs to be planned to analyze and establish the association between transaminases and the severity of dengue infection.

## CONCLUSIONS

The prevalence of transaminitis among patients with dengue fever in a tertiary care centre was found to be lower than other studies done in similar settings.
